# Robotic versus Open Gastrectomy for Gastric Cancer: A Meta-Analysis

**DOI:** 10.1371/journal.pone.0081946

**Published:** 2013-12-03

**Authors:** Guixiang Liao, Jiarong Chen, Chen Ren, Rong Li, Shasha Du, Guozhu Xie, Haijun Deng, Kaijun Yang, Yawei Yuan

**Affiliations:** 1 Department of Radiation Oncology, Nanfang Hospital, Southern Medical University, Guangzhou, Guangdong, P.R. China; 2 Department of General Surgery, Nanfang Hospital, Southern Medical University, Guangzhou, Guangdong, P.R. China; 3 Department of Neurosurgery, Nanfang Hospital, Southern Medical University, Guangzhou, Guangdong, P.R. China; University Hospital Heidelberg, Germany

## Abstract

**Aim:**

To evaluate the safety and efficacy of robotic gastrectomy versus open gastrectomy for gastric cancer.

**Methods:**

A comprehensive search of PubMed, EMBASE, Cochrane Library, and Web of Knowledge was performed. Systematic review was carried out to identify studies comparing robotic gastrectomy and open gastrectomy in gastric cancer. Intraoperative and postoperative outcomes were also analyzed to evaluate the safety and efficacy of the surgery. A fixed effects model or a random effects model was utilized according to the heterogeneity.

**Results:**

Four studies involving 5780 patients with 520 (9.00%) cases of robotic gastrectomy and 5260 (91.00%) cases of open gastrectomy were included in this meta-analysis. Compared to open gastrectomy, robotic gastrectomy has a significantly longer operation time (weighted mean differences (WMD) =92.37, 95% confidence interval (CI): 55.63 to 129.12, P<0.00001), lower blood loss (WMD: -126.08, 95% CI: -189.02 to -63.13, P<0.0001), and shorter hospital stay (WMD = -2.87; 95% CI: -4.17 to -1.56; P<0.0001). No statistical difference was noted based on the rate of overall postoperative complication, wound infection, bleeding, number of harvested lymph nodes, anastomotic leakage and postoperative mortality rate.

**Conclusions:**

The results of this meta-analysis suggest that robotic gastrectomy is a better alternative technique to open gastrectomy for gastric cancer. However, more prospective, well-designed, multicenter, randomized controlled trials are necessary to further evaluate the safety and efficacy as well as the long-term outcome.

## Introduction

Minimally invasive surgery has become widely applied in the field of general surgery including gastric cancer [1]. In 1997, robotic surgery systems were introduced as an effort to overcome technical disadvantages of laparoscopic surgery [2]. Robotic systems have 3-D imaging, tremor filter, and articulated EndoWrist (Intuitive Surgical Inc., Sunnyvale, CA, USA). With these advanced equipments, robotic surgery is superior to conventional laparoscopic surgery due to its significant improvements in visibility and manipulation [3]. Moreover, robotic gastrectomy (RG) can precisely perform lymph node dissection for gastric cancer and provide a convenient and comfortable environment for surgeons [4].

A variety of reports have demonstrated the safety and feasibility of this approach [5,6]. However, the feasibility and safety between RG and open gastrectomy (OG) in treating gastric cancer is not well elucidated. Previous reports were all based on single-institutional experience, and evidence in the context of randomized controlled trial is not available. The aim of this study is to perform a systematic review and meta-analysis of studies comparing the safety and efficacy of RG versus OG in treating gastric cancer. 

## Materials and Methods

A comprehensive search was conducted by two authors (LGX and CJR) in May 3, 2013 and updated in July 3, 2013, without restriction to regions and the date of publication. Relevant articles comparing RG and OG for gastric cancer were identified by searching PubMed, EMBASE, Web of Knowledge databases and the Cochrane Library. The following search terms were employed: robotic surgery, da Vinci, gastric cancer, gastrectomy. Gastrectomy included distal gastrectomy, proximal gastrectomy and radical gastrectomy. We excluded conference abstracts, reviews, case reports, non-comparative studies, non-relevant topic papers, non-English papers and animal studies. Relevant data from included studies were extracted and summarized by two independent authors. Any disagreements were resolved though discussions among the author group.

## Results

The outcomes that were analyzed and compared between robotic and open approaches to gastrectomy included operative time, blood loss, overall postoperative complication rate, postoperative hospital stays, numbers of harvested lymph nodes and postoperative mortality. In addition, in terms of postoperative complication, anastomotic leakage, bleeding, as well as wound infection were also analyzed.

### Quality Assessment

The methodological quality of retrospective studies was assessed by the modified Newcastle-Ottawa scale (http://www.ohri.ca/programs/clinical_epidemiology/oxford.asp). The quality of the studies assessment consisted of three items: patient selection, comparability of RG and OG groups, and exposure according to a previous meta-analysis [7].

### Statistical analysis

We performed statistical analysis by Revman software, version 5.2 (Cochrane Collaboration, Oxford, UK).Continuous and dichotomous variables were analysis by weighted mean differences (WMD) and odds ratios (OR), respectively. A 95% confidence interval (CI) was recorded. Heterogeneity among the studies was assessed using the χ^2^ test and I^2^. A fixed effect model was applied when I^2^ <50%, and a random effect model when I^2^ was greater than 50%. *P* values of less than 0.05 were considered to indicate statistical significance. Publication bias was analyzed by funnel plots and evaluated by the Begg's and Egger's test.

### Study Characteristics

**Figure 1:**
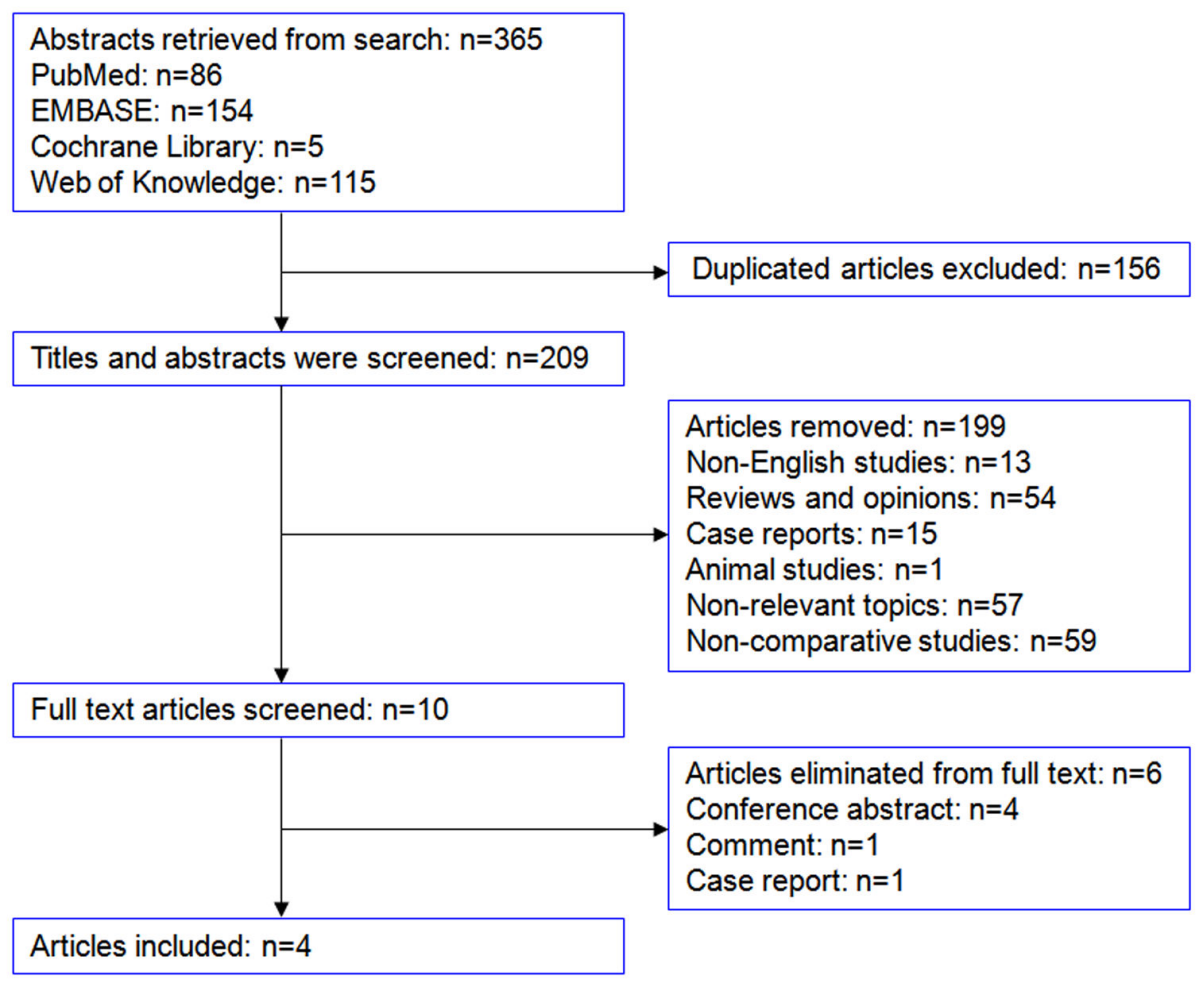
Flow chart of selection.

A total of 365 abstracts were identified from searching in PubMed, EMBASE, Cochrane Library, and Web of Knowledge electronic database. 156 duplicates were removed by using the Endnote software. After reviewing 209 titles and abstracts, 199 studies were excluded. One comment, one case report and four conference abstracts were screened among the remaining 10 studies by full articles review. Finally, four retrospective studies [4,8–10] with 5780 cases were included in our meta-analysis (Figure 1). The baseline characters of the include studies and quality assessment were listed in Table 1.

**Table 1:** Baseline characters of include studies and quality assessment (mean ± SD).

Author	Year	country	Study type	group	N	Sex	BMI	Age	Quality
						(m/f)	(mean±SD)	(mean±SD)	assessment
Caruso S[8]	2011	Italy	retrospective	RG	29	18/11	27±3	64.8±12.4	6 stars
			study	OG	120	65/55	28±4	65.1±11	
Huang KH[4]	2012	China	retrospective	RG	39	19/20	24.2±3.7	65.1±15.9	5 stars
			study	OG	586	406/180	23.7±3.6	67.9±30.1	
Kim KM[9]	2012	Korea	retrospective	RG	436	265/171	23.6±3.1	54.2±12.5	5 stars
			study	OG	4542	3008/1534	23.8±8.0	57.7±11.8	
Kim MC[10]	2010	Korea	retrospective	RG	16	10/6	21.3±3.4	53.8±15.6	6 stars
			study	OG	12	9/3	25.2±1.9	56.0±12.4	

### Operation time

**Figure 2:**
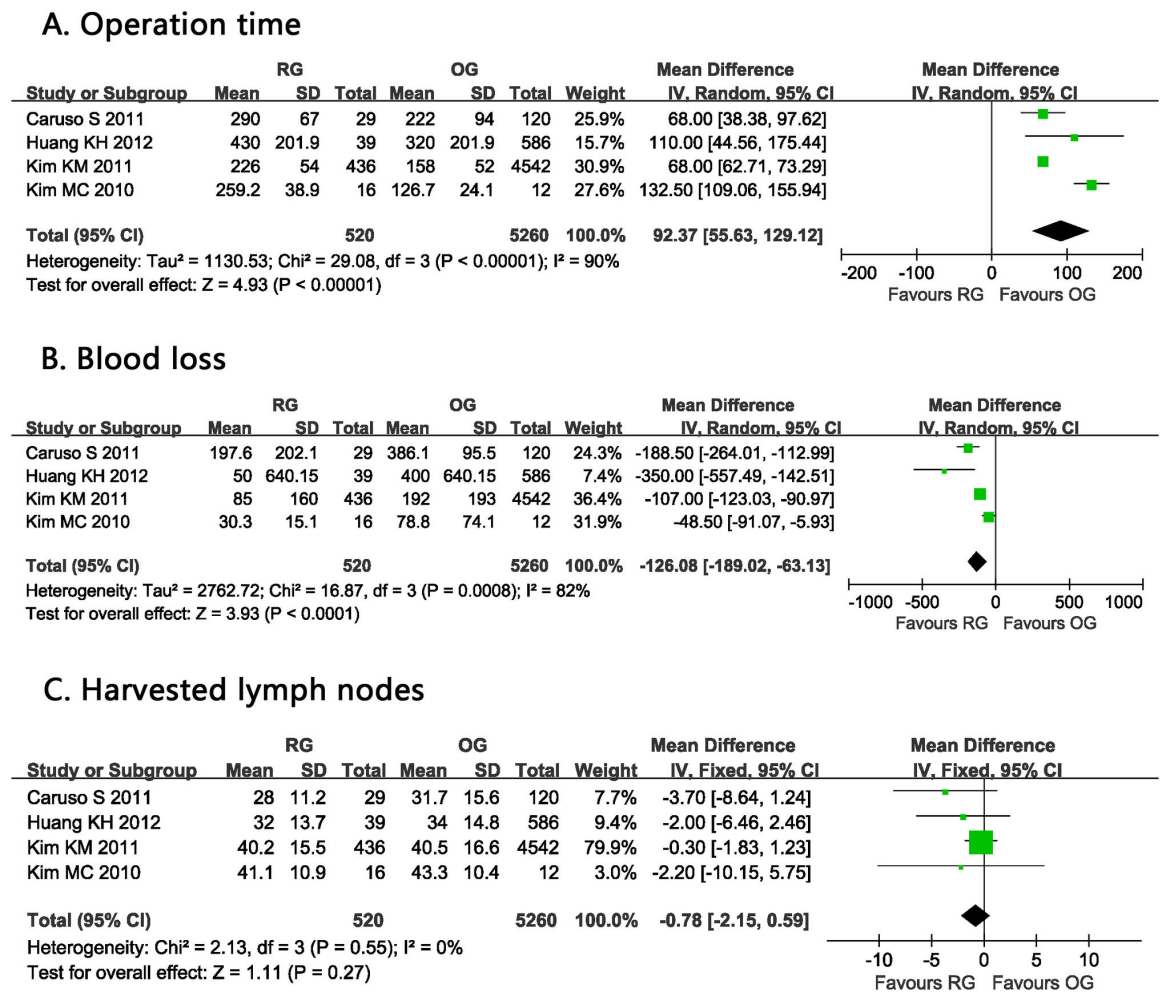
Forest plot showing a meta-analysis for robotic gastrectomy versus open gastrectomy on A. Operation time; B. Blood loss; C. Harvested lymph nodes.

Operation time was significantly longer with RG than OG reported in all included studies [4,8–10]. Pooled analysis of operation time had a significant difference between RG and OG in this regard and with a significant heterogeneity (WMD: 92.37 min, 95% CI: 55.63 to 129.12 min, *P* <0.00001, I^2^=90%) (Figure 2A).

### Blood loss

 A statistical difference of blood loss was observed between these two approaches [4,8–10]. The estimated intraoperative blood loss was significantly lower in the RG group than in the OG group. (WMD:-126.08 ml, 95% CI: -189.02 to -63.13 ml, *P* <0.0001, I^2^=82%) (Figure 2B).

### Harvested lymph nodes

The pooled data from these four studies showed no difference in the number for the harvested lymph nodes between RG and OG [4,8–10]. (WMD = -0.78; 95% CI -2.15 to 0.59; *P* =0.27) (Figure 2C).

### Postoperative hospital stay

**Figure 3:**
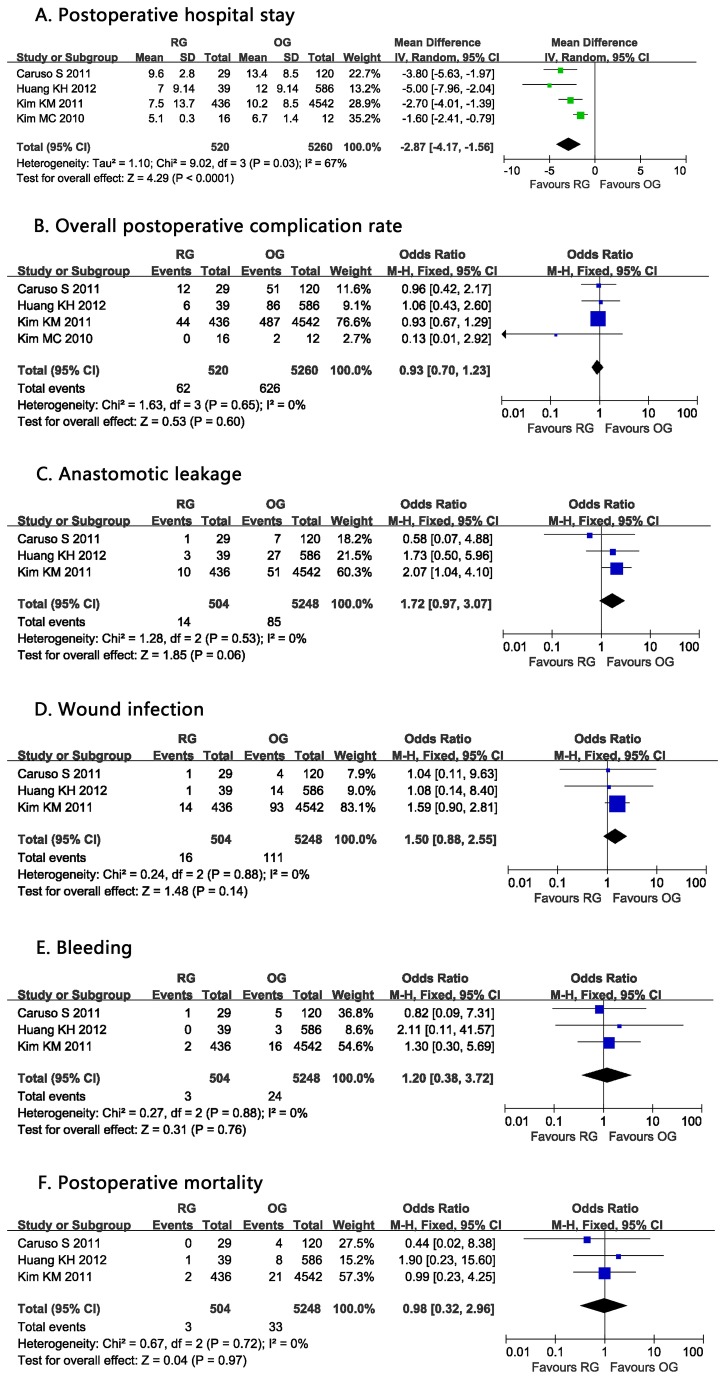
Forest plot showing a meta-analysis for robotic gastrectomy versus open gastrectomy on A. Postoperative hospital stay; B. Overall postoperative complication rate; C. Anastomotic leakage; D. Wound infection; E. Bleeding; F. Postoperative mortality.

Postoperative hospital stay was shorter with RG [4,8–10]. Compared to OG, RG reduced postoperative stay by a mean of 2.87 days. (WMD = -2.87 d; 95% CI -4.17 to -1.56 d; *P* <0.0001), with high heterogeneity among these studies (I^2^ = 67%) (Figure 3A).

### Overall postoperative complication rate

All four included studies reported postoperative complication rate [4,8–10]. The overall postoperative complication morbidity was 11.92% (62/520) in RG and 11.90% (626/5260) in OG. Meta-analysis found no significant difference (OR: 0.93, 95% CI: 0.70 to 1.23, *P* =0.60, I^2^=0%) (Figure 3B). 

### Anastomotic leakage

The rate of anastomotic leakage was described in three studies [4,8,9]. No difference was observed in pooled analysis between 2.78% (14/504) for RG and 1.62% (85/5248) for OG (OR:1.72, 95% CI: 0.97 to 3.07, P =0.06, I^2^=0%) (Figure 3C).

### Wound infection

Three studies described postoperative wound infection [4,8,9] and there were no significant differences between RG and OG. (OR: 1.50, 95% CI: 0.88 to 2.55, P=0.14, I^2^=0%) (Figure 3D).

### Bleeding

The incidence of bleeding was 0.6% in RG group and 0.4% in OG group. No differences were observed in three studies [4,8,9] (OR: 1.20, 95% CI: 0.38 to 3.72, *P* =0.76, I^2^=0%) (Figure 3E). 

### Postoperative mortality

Postoperative mortality was mentioned in three of the studies. The study by Caruso S et al. found no difference between RG and OG on 30-day mortality [8], which was similar to the results reported by Huang KH et al. [4] and Kim KM et al. [9]. Pooled analysis revealed no statistical difference with no heterogeneity (OR=0.98, 95% CI: 0.32 to 2.96, *P*=0.97, I^2^=0%) (Figure 3F).

### Publication bias

**Figure 4:**
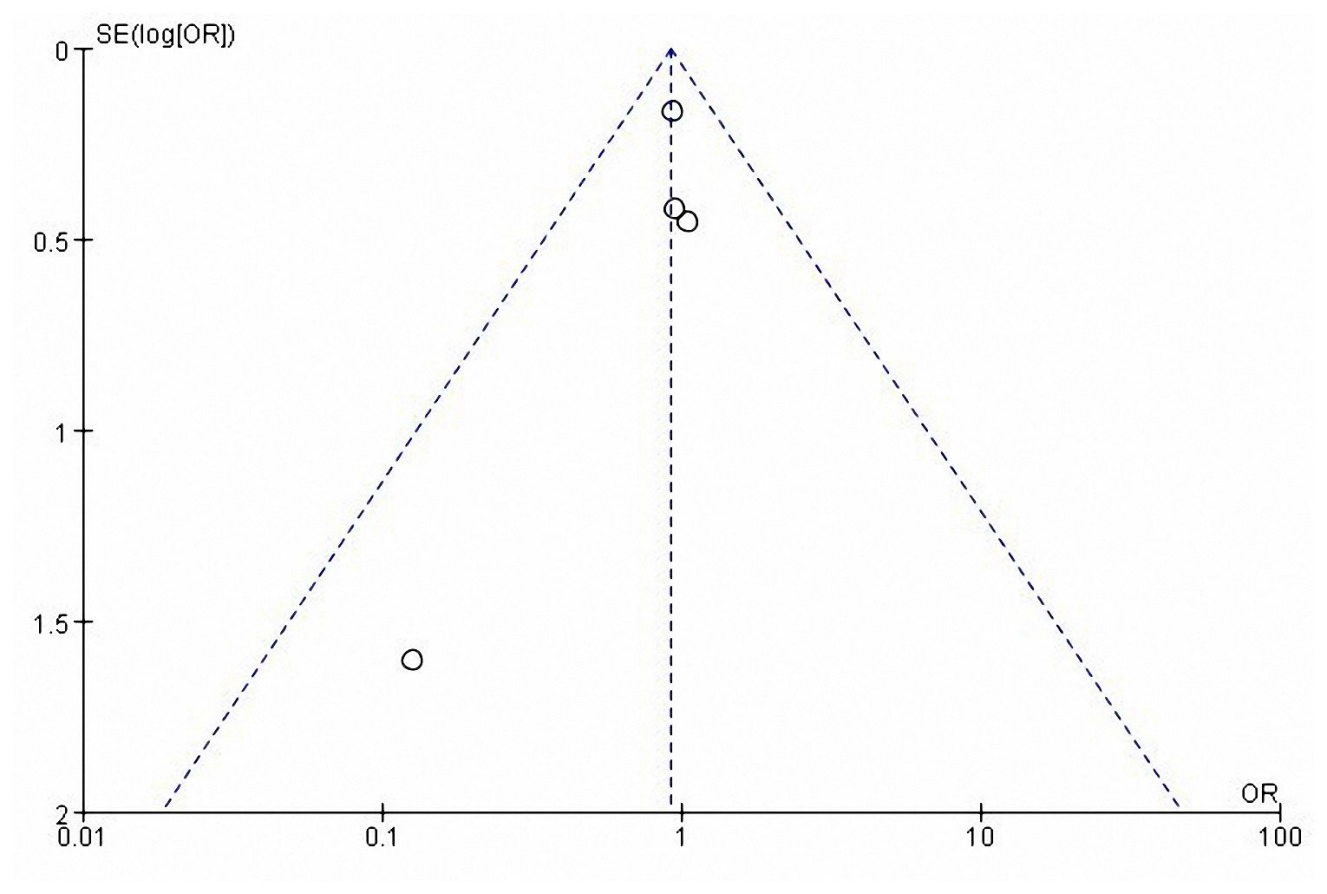
Funnel plot of overall postoperative complication rate in patients between RG and OG. OR, odds ratio; SE, standard error.

The complication rate was evaluated with a standard-error based funnel plot using fix effect size between RG and OG. The outcomes of all the studies were within the 95% CIs and were slightly unsymmetrical (Figure 4). No evidence of publication bias was revealed among these studies from statistical tests (Begg's test *P* =0.734; Egger's test *P* =0.309). 

### Sensitivity analysis

Sensitivity analysis was performed by excluding the study reported by Kim MC et al. [10] in which the total sample size was less than 50. All variables were conducted for sensitivity analysis. The index would be excluded for further sensitivity if there were not enough available studies (less than 2). The results were not significantly influenced by sensitivity analysis as shown in Table 2.

**Table 2:** Sensitivity analysis of outcomes.

Outcomes	Number of Studies	Patients	WMD/OR	95% CI	*P*	Heterogeneity	
						I^2^ (%)	*P*
Operative time (min)	3[4,8,9]	RG=504	68.26	63.07, 73.45	<0.00001	0	0.46
		OG=5428					
Postoperative hospital stay (d)	3[4,8,9]	RG=504	-3.29	-4.30, -2.29	<0.00001	15	0.31
		OG=5428					
Estimated blood loss (ml)	3[4,8,9]	RG=504	-173.88	-270.68, -77.08	0.0004	79	0.009
		OG=5428					
Total postoperative complication	3[4,8,9]	RG=504	0.95	0.71, 1.26	0.72	0	0.97
		OG=5248					
Harvested lymph nodes	3[4,8,9]	RG=504	-0.73	-2.13,0.66	0.30	0	0.37
		OG=5248					

## Discussion

With the development of technology, robot-assisted laparoscopy has been widely performed in the field of urology [11], gynecology [12] and general surgery [13], and has become an attractive option for surgeons. RG has been considered as a potentially feasible and safe technique, which has been widely reported by many studies. This meta-analysis was conducted in an attempt to evaluate the currently available evidence for the role of robotic versus open gastrectomy for gastric cancer and to identify whether the use of robotic surgery can be practically beneficial. Four studies involving 5780 patients with 520 (9.00%) cases of robotic gastrectomy and 5260 (91.00%) cases of open gastrectomy were included in this meta-analysis.

The operation time was significantly longer with RG than OG (*P* <0.00001). This could be attributed to the docking time and preparation time for RG. A previous study has reported that the mean docking time in RG was 63.3 minutes [5]. With experience gained in robotic surgery, the docking time could be reduced by half an hour [4]. Another explanation was that RG needed a learning curve in order to be proficient [14], cases with initial experience of RG may take longer than the subsequent cases due to less skilled performance. Operation time would be strikingly reduced by experience accumulated surgeons [15]. However, some studies included in this analysis also obtained cases with initial experience of RG [4]. Moreover, the operation time can be reduced by the upgraded robotic instruments. 

The most striking finding was the reduction of blood loss in RG versus OG, with statistical significance (*P* <0.0001). Due to the benefits of dexterity of scale motion and 3D image, robotic surgery can perform in a precise way while minimizing blood loss [10]. The median volume of blood loss was 30ml when performing RG reported by a previous study [16]. The lower blood loss indicated a lower transfusion rate. In addition, the amount of blood loss and the need for transfusions had a positive correlation with perioperative mortality and morbidity [17,18] . Studies have reported that a lower blood loss may result in a lower recurrence and thus, may improve the quality of life of gastric patients [19].

RG was associated with significantly shorter hospital stay (*P* <0.0001). This might be attributed to the advantages of robotic surgery systems. Robotic surgery is a minimally invasive technique [20] which contributes to reduced pain, quicker return to oral intake, as well as avoiding the long abdominal incision of open surgery and reducing tissue injury.

There was no significant difference on overall postoperative complication rate. The incidence of postoperative complication for RG (11.92%) was similar to OG (11.90%). Besides, no significant difference was observed in terms of postoperative mortality. These results demonstrated that RG is a safer and a more feasible alternative technique to OG.

Anastomotic leakage is a major complication after gastric cancer surgery [21]. The rate of anastomotic leakage ranged from 1%-10% according to previous reports [22,23]. However, according to this meta-analysis, the incidence of leakage was not significantly different between these two groups. The anastomotic leakage rate was 2.78% (14/504) for RG and 1.62% (85/5248) for OG (*P*=0.06). Yoon HM et al. reported there was no anastomotic leakage when performing RG in 36 patients [24]. As anastomotic leakage was associated with morbidity and mortality, more attention should be paid to this issue and more effort should be done to prevent leaks when performing RG. In addition, the safety of RG should be further investigated by well designed randomized controlled trials and the application of this novel approach should be with caution considering the high rate of leak when performing RG. 

No statistically difference was observed between RG and OG regarding to wound infection and bleeding.

The prognosis of gastric cancer is poor, lymph node metastasis is considered to be an important prognostic factor [25]. Previous studies reported the incidence of lymph nodes metastases in early gastric cancer ranged from 3% to 25% and the rate varied from 3%-5% in mucosa cancers and 16%-25% in submucosal tumors respectively [26]. Thus, extended lymph nodes dissection and the number of harvested lymph nodes could be used to evaluate the oncologic adequacy. For most resectable gastric cancer, the recommended standard surgery is total and distal gastrectomy with D2 lymphadenectomy [27] .Therefore, D2 lymphadenectomy is a critical part of the minimally invasive gastrectomy procedure. However, laparoscopic D2 gastrectomy entails the removal of node stations along the celiac trunk, left gastric artery, and hepatic pedicle. The technical difficulty of D2 gastrectomy has limited its pervasive application [28] .With the technical advantages, robotic surgery can achieve meticulous dissection, even in difficult lymphatic stations around major vessels or in difficult area [8]. Analysis of the pooled data revealed that the number of harvested lymph nodes was similar between RG and OG, which indicated RG could be performed safely. Several studies have also demonstrated robotic lymph node dissection was feasible and safe [29,30]. In addition, with advantages of clear 3D image and dexterity, RG could carry out a safe and effective lymphadenectomy with less blood loss [31].

Several limitations should be considered in this meta-analysis. Firstly, all the included studies are retrospective studies which are non-randomized instead of randomized controlled trials. However, according to a previously published study, well designed non-randomized comparative studies of surgical techniques can reach available results as randomized controlled trials [32]. Secondly, as is known to all, surgical parameters might be influenced by surgeon’s learning curve. In this meta-analysis, the robotic cohorts from most if not all of these institutions represented their initial experiences, which could introduce a bias against the robotic outcomes. Thirdly, high heterogeneity was existed in terms of operation time, blood loss and postoperative hospital stay. Since it was difficult to match baseline characters in all selected studies, we used a random effected model to evaluate these parameters. Fourthly, the long-term outcomes cannot be accessed because of the insufficient data. The long-term outcomes after gastrectomy were reported in only one study [8] with the follow-up time ranged from 4-53 months for RG and from 1-115months for OG. The result indicated no significant difference in survival rate between RG and OG. Finally, the cost effective between RG and OG was not compared in this meta-analysis due to insufficient data. Thus, further comparison studies addressing cost effective are needed to clarify this issue.

In conclusion, RG is safe and efficient. RG is associated with a longer operation time, less blood loss, and shorter hospital stay compared to those of OG. There is no difference on overall postoperative complication, wound infection, bleeding, anastomotic leakage rate and harvested lymph nodes. RG may be a more practical and feasible alternative technique to OG. However, more prospective, well-designed, multicenter, randomized controlled trials are necessary to further address the safety and efficacy as well as the long-term outcome of RG.

## Supporting Information

Checklist S1 **PRISMA Checklist.**
(DOCX)
