# Supplementary material for: Robotic versus Open Gastrectomy for Gastric Cancer: A Meta-Analysis
Source: PLoS One. 2013 Dec 3;8(12):e81946. doi: 10.1371/journal.pone.0081946 (PMC3849388; doi:10.1371/journal.pone.0081946)
Supplement: Checklist S1 — PRISMA Checklist. (DOCX) [file pone.0081946.s001.docx]

| **Section/topic** | **#** | **Checklist item** | **Reported on page #** |
| --- | --- | --- | --- |
| **TITLE** | | |  |
| Title | 1 | Robotic versus Open Gastrectomy for Gastric Cancer: A Meta-analysis | Title |
| **ABSTRACT** | | |  |
| Structured summary | 2 | Aim: To evaluate the safety and efficacy of robotic gastrectomy versus open gastrectomy for gastric cancer.  Methods: A comprehensive search of PubMed, EMBASE, Cochrane Library, and Web of Knowledge was performed. Systematic review was carried out to identify studies comparing robotic gastrectomy and open gastrectomy in gastric cancer. Intraoperative and postoperative outcomes were also analyzed to evaluate the safety and efficacy of the surgery. A fixed effects model or a random effects model was utilized according to the heterogeneity.  Results: Four studies involving 5780 patients with 520 (9.00%) cases of robotic gastrectomy and 5260 (91.00%) cases of open gastrectomy were included in this meta-analysis. Compared to open gastrectomy, robotic gastrectomy has a significantly longer operation time (weighted mean differences (WMD) =92.37, 95% confidence interval (CI): 55.63 to 129.12, P<0.00001), lower blood loss (WMD: -126.08, 95% CI: -189.02 to -63.13, P<0.0001), and shorter hospital stay (WMD = -2.87; 95% CI: -4.17 to -1.56; P<0.0001). No statistical difference was noted based on the rate of overall postoperative complication, wound infection, bleeding, number of harvested lymph nodes, anastomotic leakage and postoperative mortality rate.  Conclusions: The results of this meta-analysis suggest that robotic gastrectomy is a better alternative technique to open gastrectomy for gastric cancer. However, more prospective, well-designed, multicenter, randomized controlled trials are necessary to further evaluate the safety and efficacy as well as the long-term outcome. | Abstract |
| **INTRODUCTION** | | |  |
| Rationale | 3 | Minimally invasive surgery has become widely applied in the field of general surgery including gastric cancer. In 1997, robotic surgery systems were introduced, in an effort to overcome technical disadvantages of laparoscopic surgery. Robotic surgery is superior to conventional laparoscopic surgery due to its significant improvements in visibility and manipulation. Robotic gastrectomy (RG) can precisely performed lymph node dissection for gastric cancer and provided a convenient and comfortable environment for surgeons. A variety of reports have demonstrated the safety and feasibility of this approach. | Introduction |
| Objectives | 4 | The aim of this study is to perform a systematic review and meta-analysis of studies comparing the safety and efficacy of robotic gastrectomy versus open gastrectomy in treating gastric cancer. | Introduction |
| **METHODS** | | |  |
| Protocol and registration | 5 | none | n/a |
| Eligibility criteria | 6 | We included published articles comparing RG and OG for gastric cancer. We excluded conference abstracts, reviews, case reports, and non-comparative studies, non-relevant topic papers, non-English papers and animal studies. | Materials and Methods |
| Information sources | 7 | Relevant articles published to compare RG and OG for gastric cancer were identified by searching PubMed, EMBASE, Web of Knowledge databases and the Cochrance Library. | Materials and Methods |
| Search | 8 | Pubmed  #1 tumor[tiab] OR tumors[tiab] OR tumour*[tiab] OR carcinoma*[tiab] OR oncolog*[tiab] OR cancer[tiab] OR neoplas*[tiab]  #2 "Neoplasms"[Mesh]  #3 #1 or #2  #4 digest* OR gastri* OR epigastr* OR stomach*  #5 #3 and #4  #6 "Stomach Neoplasms"[Mesh] OR "Abdominal Neoplasms"[Mesh]  #7 #5 or #6  #8 "Gastrectomy"[Mesh]  #9 gastrectom*[tiab]  #10 gastri*[tiab] AND resect*[tiab]  #11 stomach[tiab] AND resect*[tiab]  #12 #8 OR #9 OR #10 OR #11  #13 "Robotics"[Mesh]  #14 surger*[tiab] AND robot* [tiab]  #15 “da vinci” OR “davinci”  #16 Aesop  #17 Zeus  #18 #13 OR #14 OR #15 OR #16 OR #17  #19 #7 AND #12 AND #18  EMBASE  #1 carcin* OR cancer* OR neoplas* OR tumour* OR tumor* OR cyst* OR growth* OR adenocarcin* OR malig*  #2 ‘neoplasm’/exp or ‘tumor’/exp  #3 #1 or #2  #4 digest* OR gastr* OR gut* OR epigastr* OR stomach*  #5 #3 and #4  #6 'stomach tumor'/exp OR 'abdominal tumor'/exp OR 'stomach cancer'/exp OR 'abdominal cancer'/exp OR 'stomach carcinoma'/exp OR 'stomach carcinogenesis'/exp OR 'stomach carcinoid'/exp  #7 #5 or #6  #8 'gastrectomy'/exp OR gastrectomy  #9 gastrectom*:ab,ti  #10 (gastr* NEAR/10 resect*):ab,ti  #11 (stomach NEAR/10 resect*):ab,ti  #12 #8 OR #9 OR #10 OR #11  #13 'robotics'/exp OR robotics  #14 (surger* AND robot*):ab,ti  #15 'da vinci' OR ‘davinci’  #16 Aesop  #17 Zeus  #18 #13 OR #14 OR #15 OR #16 OR #17  #19 #7 AND #12 AND #18 | Materials and Methods |
| Study selection | 9 | 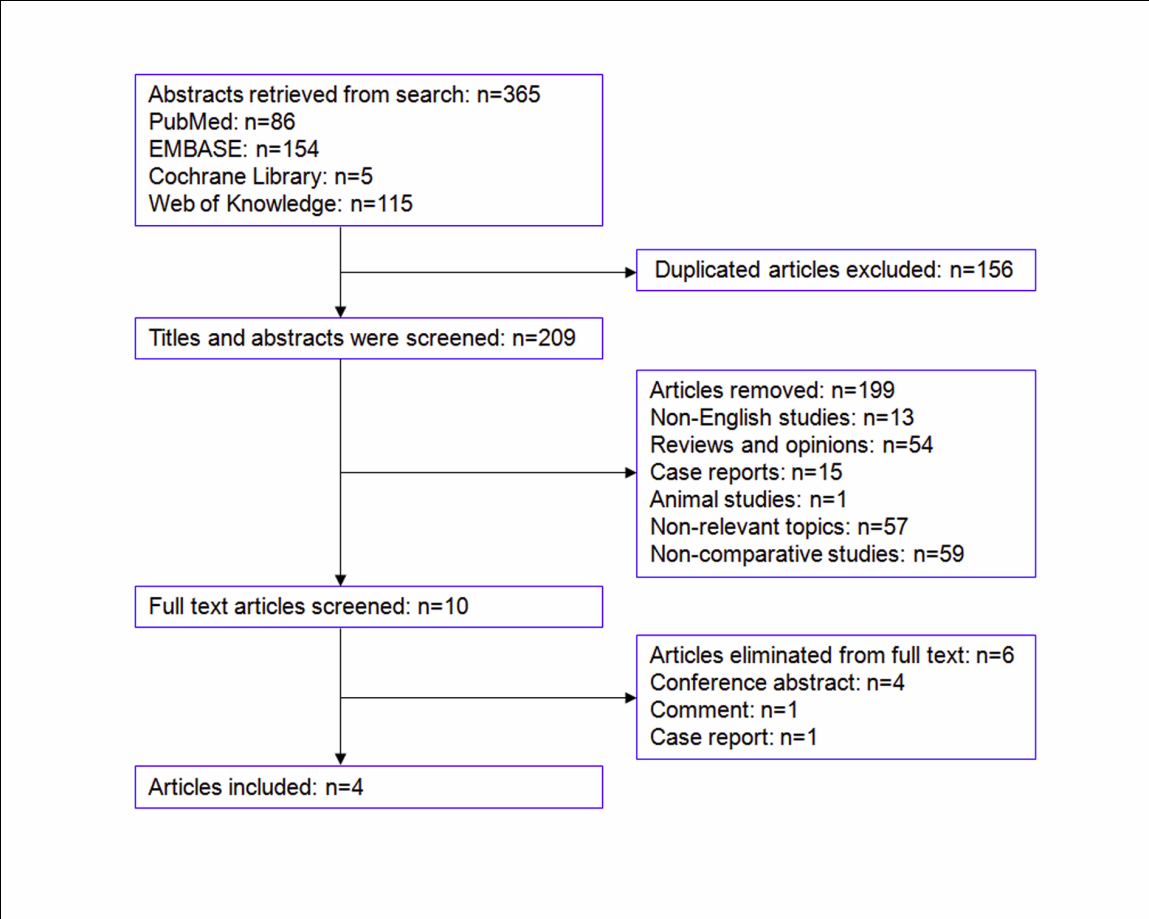 | Results |
| Data collection process | 10 | Relevant data from included studies were extracted and summarized by two independent authors. | Materials and Methods |
| Data items | 11 | Basic characters of included study   \| Author \| Year \| country \| Study type \| group \| N \| Sex  m/f \| BMI  Mean \| Age  Mean \| Quality assessment \| \| --- \| --- \| --- \| --- \| --- \| --- \| --- \| --- \| --- \| --- \| \| Caruso S \| 2011 \| Italy \| retrospective study \| RG  OG \| 29  120 \| 18/11  65/55 \| 27±3  28±4 \| 64.8±12.4  65.1±11 \| 6 stars \| \| Huang KH \| 2012 \| China \| retrospective study \| RG  OG \| 39  586 \| 19/20  406/180 \| 24.2±3.7  23.7±3.6 \| 65.1±15.9  67.9±30.1 \| 5 stars \| \| Kim KM \| 2012 \| Korea \| retrospective study \| RG  OG \| 436  4542 \| 265/171  3008/1534 \| 23.6±3.1  23.8±8.0 \| 54.2±12.5  57.7±11.8 \| 5 stars \| \| Kim MC \| 2010 \| Korea \| retrospective study \| RG  OG \| 16  12 \| 10/6  9/3 \| 21.3±3.4  25.2±1.9 \| 53.8±15.6  56.0±12.4 \| 6 stars \|   Data of interest [mean (SD)]   \| Author \| year \| group \| N \| Operation time \| Blood loss \| Harvested lymph nodes \| Postoperative hospital stay \| \| --- \| --- \| --- \| --- \| --- \| --- \| --- \| --- \| \| Caruso S \| 2011 \| RG \| 29 \| 290(67) \| 197.6(202.1) \| 28(11.2) \| 9.6(2.8) \| \| OG \| 120 \| 222(94) \| 386.1(95.5) \| 31.7(15.6) \| 13.4(8.5) \| \| Huang KH \| 2012 \| RG \| 39 \| 430(201.9) \| 50(640.15) \| 32(13.7) \| 7(9.14) \| \| SG \| 586 \| 320(201.9) \| 400(640.15) \| 34(14.8) \| 12(9.14) \| \| Kim KM \| 2012 \| RG \| 436 \| 226(54) \| 85(160) \| 40.2(15.5) \| 7.5(13.7) \| \| OG \| 4542 \| 158(52) \| 192(193) \| 40.5(16.6) \| 10.2(8.5) \| \| Kim MC \| 2010 \| RG \| 16 \| 259.2(38.9) \| 30.3(15.1) \| 41.1(10.9) \| 5.1(0.3) \| \| OG \| 12 \| 126.7(24.1) \| 78.8(74.1) \| 43.3(10.4) \| 6.7(1.4) \|   Data of interest   \| Author \| year \| group \| N \| overall postoperative complication \| Anastomosis leakage \| Wound infection \| bleeding \| Postoperative  mortality \| \| --- \| --- \| --- \| --- \| --- \| --- \| --- \| --- \| --- \| \| Caruso S \| 2011 \| RG \| 29 \| 12 \| 1 \| 1 \| 1 \| 0 \| \| OG \| 120 \| 51 \| 7 \| 4 \| 5 \| 4 \| \| Huang KH \| 2012 \| RG \| 39 \| 6 \| 3 \| 1 \| 0 \| 1 \| \| SG \| 586 \| 86 \| 27 \| 14 \| 3 \| 8 \| \| Kim KM \| 2012 \| RG \| 436 \| 44 \| 10 \| 14 \| 2 \| - \| \| OG \| 4542 \| 487 \| 51 \| 93 \| 16 \| - \| \| Kim MC \| 2010 \| RG \| 16 \| 0 \| - \| - \| - \| - \| \| OG \| 12 \| 2 \| - \| - \| - \| - \| | Results |
| Risk of bias in individual studies | 12 | Publication bias was analysis by funnel plots.  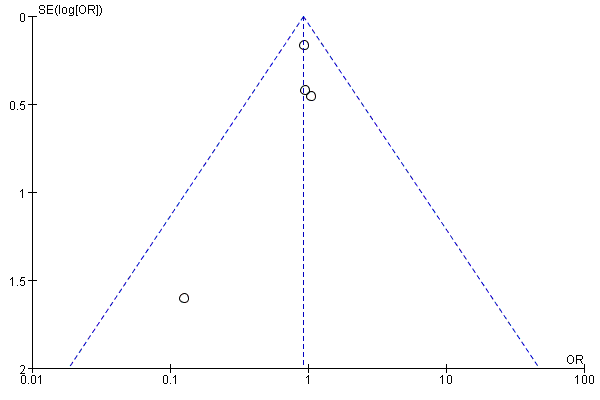 | Results |
| Summary measures | 13 | Continuous and dichotomous variables were analysis by Weighted mean differences (WMD) and odds ratios (OR), respectively. And 95% confidence interval (CI) was recorded. | Results |
| Synthesis of results | 14 | Heterogeneity among the studies was assessed using the χ^2^ test and I^2^. A fixed effect model was applied when I^2^ <50%, and a random effect model when I^2^ greater than 50%. P values of less than 0.05 were considered to indicate statistical significance. | Results |
| Risk of bias across studies | 15 | Publication of bias  Complication rate was evaluated with standard-error based funnel plot using fix effect size between RG and OG. Outcomes of all the studies were within the 95% CIs and were slightly unsymmetrical. No evidence of publication bias or heterogeneity was revealed among these studies (P =0.55). | Results |
| Additional analyses | 16 | Sensitivity analysis was performed by excluding the study reported by Kim MC et al. in which the total sample size was less than 50. All variables were conducted for sensitivity analysis. The index would be excluded for further sensitivity if there were not enough available studies (less than 2). The results were not significantly influenced by sensitivity analysis.   \| Outcomes \| Number of Studies \| Patients \| WMD/OR \| 95% CI \| *P* \| Heterogeneity \| \| \| \| --- \| --- \| --- \| --- \| --- \| --- \| --- \| --- \| --- \| \| I^2^(%) \| \| *P* \| \| Operative time (min) \| 3 \| RG=504  OG=5428 \| 68.26 \| 63.07, 73.45 \| <0.00001 \| 0 \| 0.46 \| \| \| Postoperative hospital stay (d) \| 3 \| RG=504  OG=5428 \| -3.29 \| -4.30, -2.29 \| <0.00001 \| 15 \| 0.31 \| \| \| Estimated blood loss (ml) \| 3 \| RG=504  OG=5428 \| -173.88 \| -270.68, -77.08 \| 0.0004 \| 79 \| 0.009 \| \| \| Total postoperative complication \| 3 \| RG=504  OG=5248 \| 0.95 \| 0.71, 1.26 \| 0.72 \| 0 \| 0.97 \| \| \| Harvested lymph nodes \| 3 \| RG=504  OG=5248 \| -0.73 \| -2.13,0.66 \| 0.30 \| 0 \| 0.37 \| \| | Results |
| **RESULTS** | | |  |
| Study selection | 17 | A total of 365 abstracts were identified through search in PubMed, EMBASE, Cochrane Library, and Web of Knowledge electronic database. 156 duplicates were removed by using Endnote software. After reviewing 209 titles and abstracts, 199 studies were excluded. One comment, one case report and four conference abstracts were screened among the remaining 10 studies by full articles review. Finally, four retrospective studies with 5780 cases were included in our meta-analysis.  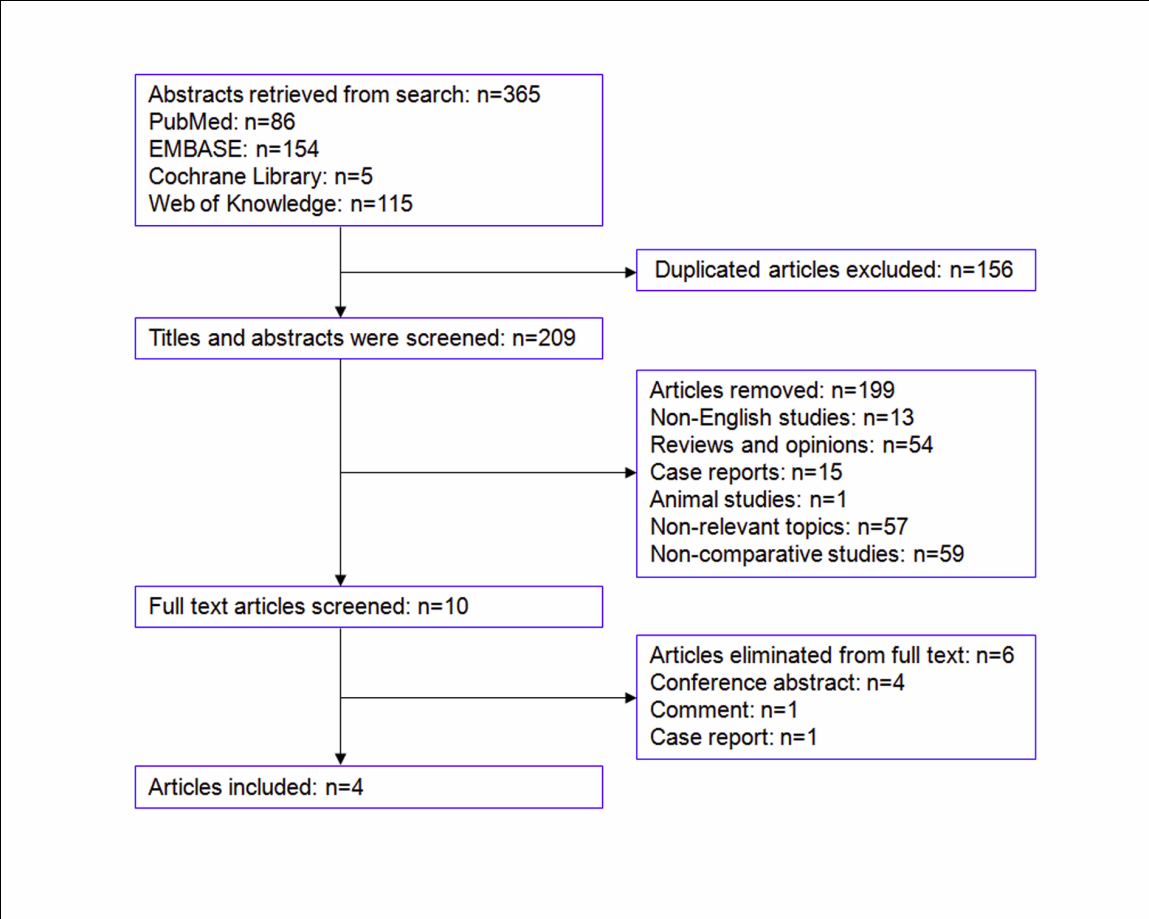 | Results |
| Study characteristics | 18 | Baseline characters of include studies and quality assessment (mean ± SD)   \| Author \| Year \| country \| Study type \| group \| N \| Sex  m/f \| BMI  Mean \| Age  Mean \| Quality assessment \| \| --- \| --- \| --- \| --- \| --- \| --- \| --- \| --- \| --- \| --- \| \| Caruso S \| 2011 \| Italy \| retrospective study \| RG  OG \| 29  120 \| 18/11  65/55 \| 27±3  28±4 \| 64.8±12.4  65.1±11 \| 6 stars \| \| Huang KH \| 2012 \| China \| retrospective study \| RG  OG \| 39  586 \| 19/20  406/180 \| 24.2±3.7  23.7±3.6 \| 65.1±15.9  67.9±30.1 \| 5 stars \| \| Kim KM \| 2012 \| Korea \| retrospective study \| RG  OG \| 436  4542 \| 265/171  3008/1534 \| 23.6±3.1  23.8±8.0 \| 54.2±12.5  57.7±11.8 \| 5 stars \| \| Kim MC \| 2010 \| Korea \| retrospective study \| RG  OG \| 16  12 \| 10/6  9/3 \| 21.3±3.4  25.2±1.9 \| 53.8±15.6  56.0±12.4 \| 6 stars \| | Results |
| Risk of bias within studies | 19 | Publication bias was analysis by funnel plots.  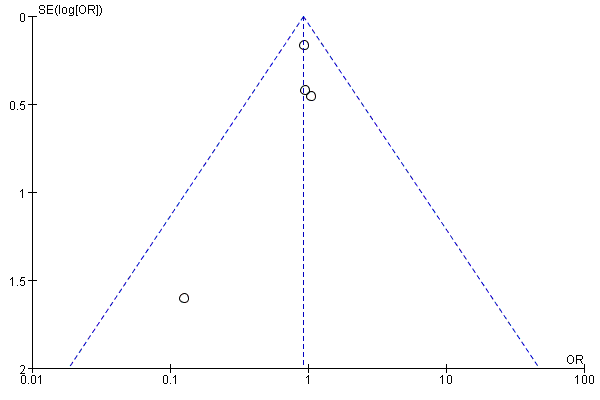 | Results |
| Results of individual studies | 20 | 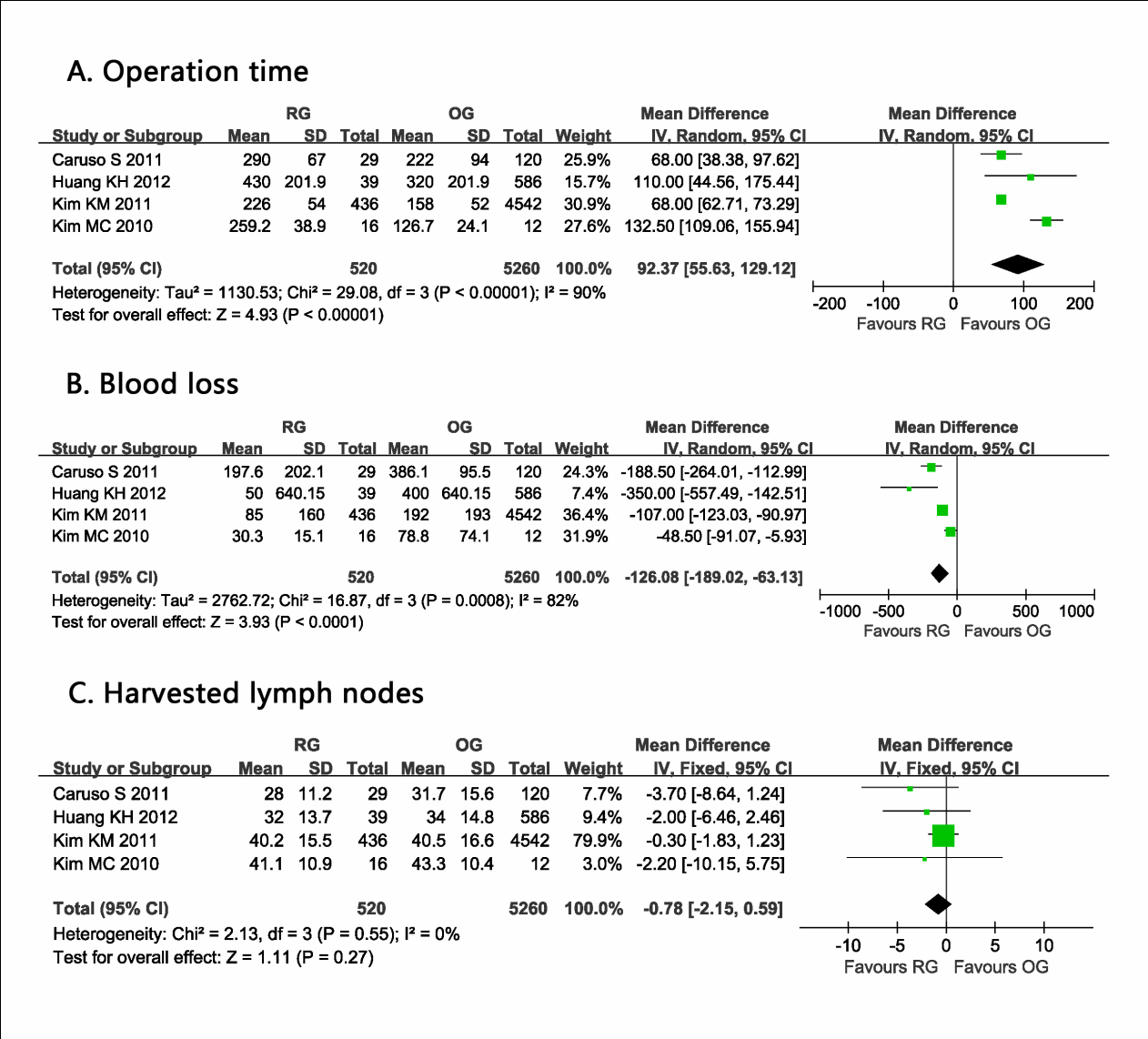  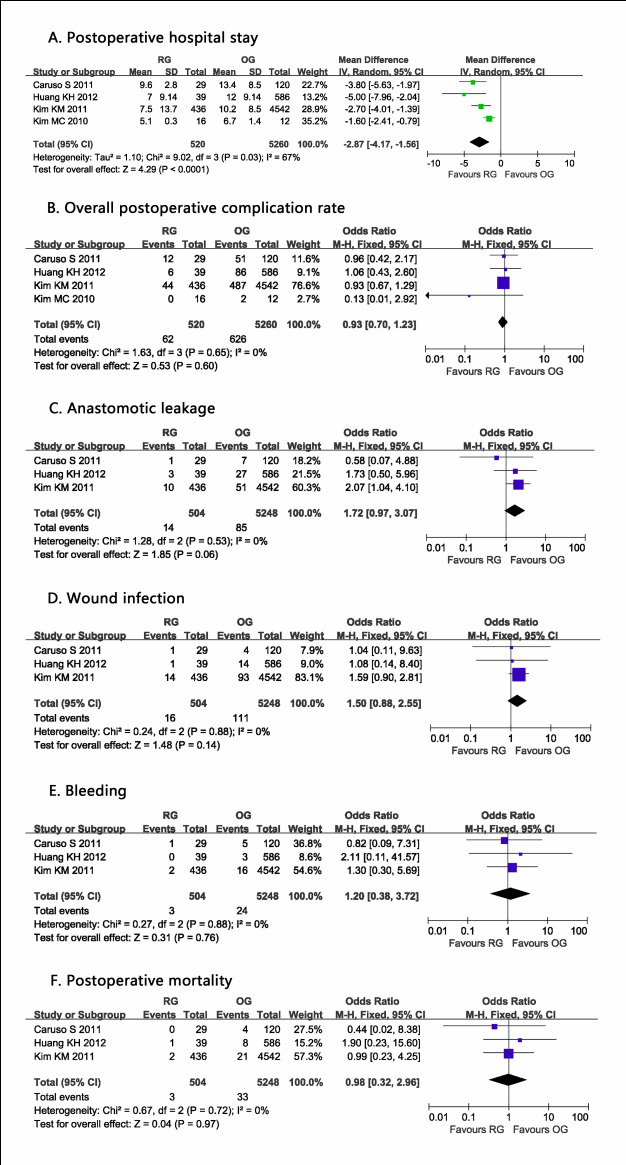 | Results |
| Synthesis of results | 21 | See item 20 | Results |
| Risk of bias across studies | 22 | See Item 15 | Results |
| Additional analysis | 23 | See Item 16 | Results |
| **DISCUSSION** | | |  |
| Summary of evidence | 24 | The operation time was significantly longer with RG than OG (P <0.00001). This could be attributed to the docking time and preparation time for RG. A previous study has reported that the mean docking time in RG was 63.3 minutes. With experience gained in robotic surgery, the docking time could be reduced by half an hour. Another explanation was that RG needed a learning curve in order to be proficient, cases with initial experience of RG may take longer than the subsequent cases due to less skilled performance. Operation time would be strikingly reduced by experience accumulated surgeons. However, some studies included in this analysis also obtained cases with initial experience of RG. Moreover, the operation time can be reduced by the upgraded robotic instruments.  The most striking finding was the reduction of blood loss in RG versus OG, with statistical significance (P <0.0001). Due to the benefits of dexterity of scale motion and 3D image, robotic surgery can perform in a precise way while minimizing blood loss. The median volume of blood loss was 30ml when performing RG reported by a previous study. The lower blood loss indicated a lower transfusion rate. In addition, the amount of blood loss and the need for transfusions had a positive correlation with perioperative mortality and morbidity. Studies have reported that a lower blood loss may result in a lower recurrence and thus, may improve the quality of life of gastric patients.  RG was associated with significantly shorter hospital stay (P <0.0001). This might be attributed to the advantages of robotic surgery systems. Robotic surgery is a minimally invasive technique which contributes to reduced pain, quicker return to oral intake, as well as avoiding the long abdominal incision of open surgery and reducing tissue injury.  There was no significant difference on overall postoperative complication rate. The incidence of postoperative complication for RG (11.92%) was similar to OG (11.90%). Besides, no significant difference was observed in terms of postoperative mortality. These results demonstrated that RG is a safer and a more feasible alternative technique to OG.  Anastomotic leakage is a major complication after gastric cancer surgery. The rate of anastomotic leakage ranged from 1%-10% according to previous reports. However, according to this meta-analysis, the incidence of leakage was not significantly different between these two groups. The anastomotic leakage rate was 2.78% (14/504) for RG and 1.62% (85/5248) for OG (P=0.06). Yoon HM et al. reported there was no anastomotic leakage when performing RG in 36 patients. As anastomotic leakage was associated with morbidity and mortality, more attention should be paid to this issue and more effort should be done to prevent leaks when performing RG. In addition, the safety of RG should be further investigated by well designed randomized controlled trials and the application of this novel approach should be with caution considering the high rate of leak when performing RG.  No statistically difference was observed between RG and OG regarding to wound infection and bleeding. | Discussion |
| Limitations | 25 | Several limitations should be considered in this meta-analysis. Firstly, all the included studies are retrospective studies which are non-randomized instead of randomized controlled trials. However, according to a previously published study, well designed non-randomized comparative studies of surgical techniques can reach available results as randomized controlled trials. Secondly, as is known to all, surgical parameters might be influenced by surgeon’s learning curve. In this meta-analysis, the robotic cohorts from most if not all of these institutions represented their initial experiences, which could introduce a bias against the robotic outcomes. Thirdly, high heterogeneity was existed in terms of operation time, blood loss and postoperative hospital stay. Since it was difficult to match baseline characters in all selected studies, we used a random effected model to evaluate these parameters. Fourthly, the long-term outcomes cannot be accessed because of the insufficient data. The long-term outcomes after gastrectomy were reported in only one study with the follow-up time ranged from 4-53 months for RG and from 1-115months for OG. The result indicated no significant difference in survival rate between RG and OG. Finally, the cost effective between RG and OG was not compared in this meta-analysis due to insufficient data. Thus, further comparison studies addressing cost effective are needed to clarify this issue. | Discussion |
| Conclusions | 26 | In conclusion, RG is safe and efficient. RG is associated with a longer operation time, less blood loss, and shorter hospital stay compared to those of OG. There is no difference on overall postoperative complication, wound infection, bleeding, anastomotic leakage rate and harvested lymph nodes. RG may be a more practical and feasible alternative technique to OG. However, more prospective, well-designed, multicenter, randomized controlled trials are necessary to further address the safety and efficacy as well as the long-term outcome of RG. | Discussion |
| **FUNDING** | | |  |
| Funding | 27 | This work was supported by a Natural Science Foundation of China grant (81272508), key applied and basic projects of Guangzhou science and technology program (11C22120714).The funders had no role in study design, data collection and analysis, decision to publish, or preparation of the manuscript. | Funding |
